# The Emerging Role of Sestrin2 in Cell Metabolism, and Cardiovascular and Age-Related Diseases

**DOI:** 10.14336/AD.2019.0320

**Published:** 2020-02-01

**Authors:** Wanqing Sun, Yishi Wang, Yang Zheng, Nanhu Quan

**Affiliations:** ^1^Cardiovascular Center, First Affiliated Hospital of Jilin University, Changchun, Jilin, China; ^2^Fuwai Hospital, National Center of Cardiovascular Diseases, Beijing, China; ^3^Department of Physiology and Pathophysiology, Fourth Military Medical University, Xi’an, Shaanxi, China

**Keywords:** sestrin2, cell metabolism, aging, cancer, myocardial ischemia

## Abstract

Sestrins (Sesns), including Sesn1, Sesn2, and Sesn3, are cysteine sulfinyl reductases that play critical roles in the regulation of peroxide signaling and oxidant defense. Sesn2 is thought to regulate cell growth, metabolism, and survival response to various stresses, and act as a positive regulator of autophagy. The anti-oxidative and anti-aging roles of Sesn2 have been the focus of many recent studies. The role of Sesn2 in cellular metabolism and cardiovascular and age-related diseases must be analyzed and discussed. In this review, we discuss the physiological and pathophysiological roles and signaling pathways of Sesn2 in different stress-related conditions, such as oxidative stress, genotoxic stress, and hypoxia. Sesn2 is also involved in aging, cancer, diabetes, and ischemic heart disease. Understanding the actions of Sesn2 in cell metabolism and age-related diseases will provide new evidence for future experimental research and aid in the development of novel therapeutic strategies for Sesn2-related diseases.

## 1.Introduction

Aging is an eternal theme in human life and many people seek a return to their youth, or at least a way of slowing the aging process. Aging is a complicated process, and many pathological changes are involved, including dysfunctional cell metabolism, the attenuation of host defenses, and memory loss [1-3] Consequently, aging can lead to various diseases, such as cancer, diabetes, and myopathy [4, 5]. Although much progress has been made in anti-aging research, this mysterious process is not fully understood, and new therapeutic targets are urgently needed. Aging, once considered irreversible as a major risk factor for many chronic diseases, is in fact extremely malleable. Strategies to delay and potentially reverse the ageing process have long been a focus of research even if molecular mechanisms are not fully understood. As aging progresses, individuals are less tolerant of various stresses. Notably, stress-inducible proteins have been claimed as involved in aging.

Sestrins (Sesns) are a group of highly conserved stress-inducible proteins that respond to a variety of environmental stresses including oxidative stress, DNA damage, and hypoxia [6]. Two main biological functions of Sesns have been identified. First, Sesns act as antioxidants that can decrease the accumulation of reactive oxygen species (ROS) [7]. Second, they function as inhibitors of mammalian targets of rapamycin complex 1 (mTORC1) [7, 8]. Sesns bind to kelch-like ECH-associated protein 1 (Keap1) and p62/SQSTM, and suppress the autophagic degradation of Keap1, leading to nuclear factor (erythroid-derived 2)-like 2 (Nrf2) activation [9]. Moreover, Sesns can suppress mTORC1 activity through the activation of AMPK [8, 10, 11]. Based on these functions, Sesns are thought to attenuate various age-related metabolic disorders, including insulin resistance, glucose intolerance, mitochondrial dysfunction, muscle degeneration, and cardiac dysfunction [8, 12-15]. Recent studies using Sesn2 and Sesn3 knockout (KO) mice have confirmed the role of Sesns in the suppression of age- and obesity-associated metabolic diseases [9, 12].

In this review, we summarize the latest advances regarding the association of Sesns with cardiovascular and age-related metabolic diseases. First, we review the general background of Sesn2. Then, we discuss the role of Sesn2 in several pathophysiological conditions, such as oxidative stress, genotoxic stress, and hypoxia. In addition, we illustrate several signaling pathways that are associated with Sesn2. Finally, we highlight certain Sesn2-related conditions, including aging, cancer, diabetes, and ischemic heart disease. This review will provide a better understanding of the actions of Sesn2 in cell metabolism and age-related diseases as well as new evidence for future experimental research and promote the role of Sesn2 as a novel therapeutic target.

## 2.General background of Sesn2

In 1999, Velasco-Miguel et al. first reported that *PA26*, known as *Sesn1*, was a novel target of the tumor suppressor p53 and has properties common to the growth arrest and DNA damage-inducible (GADD) family of stress-response genes, which suggests that Sesn1 acts as a potential novel regulator of cellular growth [16]. In 2002, Budanov et al. used cDNA microarray hybridization to identify novel genes that participate in cellular responses to prolonged hypoxia. They found a novel gene, *Hi95 (Sesn2)*, which shares marked homology with p53-regulated GADD family member *PA26* [17]. Increased Hi95 transcription was observed in response to prolonged hypoxia, DNA damage, and oxidative stress, but not to hyperthermia or serum starvation. Furthermore, the induction of Hi95 by prolonged hypoxia or oxidative stress is most likely p53-independent and is induced by DNA damage (gamma- or ultraviolet [UV]-irradiation, or doxorubicin) in a p53-dependent manner. They then demonstrated that the over-expression of Hi95 full-length cDNA is toxic among many types of cultured cells and leads either to apoptotic death or sensitization to serum starvation and DNA damage. However, conditional over-expression of the Hi95 cDNA in MCF7-tet-off cells was found to be protective against cell death triggered by hypoxia/glucose deprivation or H_2_O_2_. Their study suggests that Sesn2 is involved in the regulation of cell survival in response to various types of stress. In addition, Sesn2 has been suggested to be involved in the pathogenesis of heterotaxia, which is characterized by an abnormal left-right axis formation and reversed left-right polarity of one or more organ systems [18]. They found that, in a patient with heterotaxia, the PA26 gene is disrupted by the 6q21 breakpoint.

As a target of *p53*, which is the guardian of the genome, Sesn2 protein acts as an antioxidant, which is responsible for diminishing the accumulation of ROS [19] and inhibits mTORC1 signaling [8]. Both the accumulation of ROS and the activation of mTORC1 are strongly associated with aging and age-related diseases [20, 21], indicating that Sesn2 is a novel target for anti-aging research. While accumulating evidence suggests an association between Sesn2 and aging, new reports have demonstrated that Sesn2 plays a role in cancer, inflammation, and neurological diseases [22-26]. In the current review, we discuss the regulation of Sesn2 and its role in the regulation of metabolism and aging.

## 3.Regulation of Sesn2 expression under various conditions

In response to various insults, cells respond by adjusting to changing conditions to survive. For example, autophagy will occur if a cell is starved; the cell will survive to a certain extent for some time with the help of autophagy. Moreover, a cell will undergo anoxic respiration in hypoxic conditions. Through these adjustments, a cell can save energy to enable its survival under harsh conditions. The expression of Sesn2 is stress-inducible and involved in the regulation of cell viability in response to different stress conditions [17]. Therefore, it is important to elucidate the regulatory mechanism of Sesn2 expression under various types of stress.

### 3.1. Oxidative stress

Oxidative stress is characterized by the overproduction of ROS and reactive nitrogen species (RNS), which leads to dysfunction of the cell. Oxidative stress can not only oxidize DNA and RNA, but can also damage other molecules including proteins and lipids [20]. The accumulation of the products of oxidative stress causes aging [20], cell apoptosis [27], neurodegeneration [28], and metabolic syndrome [29]. The members of the Sesn family are induced by oxidative stress via different mechanisms. Sesn1 is induced in a p53-dependent manner, while Sesn2 can be induced by oxidative stress in a p53-dependent manner [30]. Recent studies have demonstrated that the induction of Sesn2 by oxidative stress is dependent on Nrf2 and activator protein-1 [31, 32]. Sesn2 activates Nrf2 by removing its inhibitor Keap1, which leads to activation of the antioxidant system. Sesn2 is induced by NMDA receptor activation in neurons in a CCAAT-enhancer-binding protein beta (c/EBPβ)-dependent manner [33]. The Cys125 residue of Sesn2, which is conserved throughout the Sesn-family, is critical for the antioxidant activity of Sesn2 [19]. Sesn2 is proven to inhibit uncoupling protein 1 expression by suppressing ROS-mediated activation of p38 mitogen-activated protein kinase in brown adipose tissue [34]. Additionally, Sesn2 may decrease renal oxidative stress and mediate the dopamine D2 receptor-induced inhibition of ROS production [35]. Therefore, Sesn2 plays a key role in maintaining cellular homeostasis during oxidative stress.

### 3.2. Genotoxic stress

Genotoxic stress is suggested to boost aging, and interfere with DNA damage repair [36]. Genotoxic stress can be triggered by the overproduction of DNA-damaging molecules, such as ROS, RNS, reactive carbonyl species, lipid peroxidation products, and DNA-alkylating agents. Excessive genotoxic stress can activate DNA damage-sensing signaling pathways, including activation of tumor suppressor p53, which confers cell cycle-inhibitory and pro-apoptotic activities to eliminate damaged cells. As a DNA damage-inducible protein, Sesn2 play a critical role in the response to genotoxic stress [37]. Sesn2 is induced upon DNA damage (gamma- and UV-irradiation and genotoxic metabolites) through the activation of p53 [16, 17]. Budanov et al. demonstrated that Sesn2 activates AMPK signaling and inhibits mTOR upon genotoxic challenge, while Sesn2-deficient mice fail to inhibit mTOR signaling [10]. The inhibition of mTOR signaling can minimize the synthesis of new proteins and membranes during genotoxic stress, which ultimately saves energy for DNA repair.

### 3.3. Hypoxia

Hypoxia, that is, insufficient oxygen availability, is another stimulus that can induce Sesn2 activation [17]. Sesn2 (Hi95) was first identified as a gene that was activated by hypoxia in human neuroblastoma cells [17]. Transcriptional activation of Sesn2 upon hypoxia is reported to be hypoxia-inducible factor-1-dependent in mouse epithelial tracheal cells [38]. A recent study indicated that Sesn2 activation is independent of p53 but requires the PI3K/Akt pathway in response to energetic stress induced by 2-deoxyglucose (2-DG) [39]. The inhibition of Akt, as well as loss of Sesn2, sensitizes cells to 2-DG-induced apoptosis. Moreover, the rescue of Sesn2 partially reverses the pro-apoptotic effects of 2-DG. In addition, metformin (inhibitor of mitochondrial respiration), induces the expression of Sesn2 via an unknown mechanism [39].

### 3.4. *Endoplasmic reticulum stress*

Endoplasmic reticulum (ER) stress is characterized by an accumulation of misfolded proteins. Upon ER stress, the attenuation of protein translation is essential for maintaining tissue homeostasis. Park et al. demonstrated that Sesn2 expression is induced by the ER stress-activated transcription factor c/EBPβ. Once induced, Sesn2 subsequently prevents protein synthesis by inhibiting mTORC1. Conversely, if Sesn2 is lacking, cells are highly susceptible to ER stress-associated cell death due to the continued translation of large amounts of protein [14]. A recent study indicated that the induction of Sesn2 under ER stress is dependent on activating transcription factor 6, and Sesn2 plays a hepatoprotective role in the presence of excess ER stress by inhibiting CCAAT-enhancer-binding protein homologous protein, c-Jun N-terminal kinase (JNK), and p38 [40]. ER stress is an aspect of the pathogenesis of aging and a variety of human diseases, such as cardiovascular diseases; however, the transcription factors involved in the induction of Sesn2 by ER stress and the protective mechanisms triggered by Sesn2 against ER stress-mediated cell death require further exploration.

## 4.Sesn2 and signaling pathways

### 4.1. Keap1/Nrf2 signaling pathway

Keap1 is a cysteine-rich protein that represses Nrf2 activity [41]. Under quiescent conditions, Nrf2 localizes in the cytoplasm and binds to Keap1 [42]. Once activated by oxidative stress, Nrf2 disassociates from Keap1, translocates to the nucleus, and binds to the antioxidant response element sequences in the promoter regions of a set of cytoprotective genes, such as hemoxygenase 1, NADPH:quinone oxidoreductase, and glutathione-S transferase, and activates their transcription [43]. In the nucleus, Nrf2 can also be degraded by the ubiquitin-proteasome system. Nrf2 activation contributes to protection against a variety of diseases, including cardiovascular and neurodegenerative diseases [44-48]. Therefore, Keap1/Nrf2 signaling plays a critical role in maintaining homeostasis in cells under oxidative stress.

It has been suggested that Sesn2, activated by oxidative stress, increases the expression of sulfiredoxin through the activation of Nrf2 [9]. In addition, Sesn2 activates Nrf2 by promoting p62-dependent autophagic degradation of Keap1, thus leading to Nrf2 activation, and decreased susceptibility of the liver to oxidative damage [9]. Sesn2 decreases the accumulation of ROS and stimulates anti-oxidant defenses via Keap1/Nrf2 signaling activation.

### 4.2. AMPK/mTORC1 signaling pathway

The target of rapamycin (TOR), an evolutionary conserved protein, is a pivotal regulator of cell growth, proliferation, metabolism, and autophagy [7]. TOR was first identified as a protein kinase inhibited by rapamycin [49-51]. Mammalian TOR (mTOR) is present in two distinct complexes, namely, mTORC1, which is sensitive to rapamycin, and mTORC2, which is not. mTORC1 consists of mTOR raptor, PRAS40, and mLST8, and is responsible for the regulation of cell growth and protein synthesis [52, 53]. mTORC2 consists of mTOR, Rictor, Sin1, Protor, and mLST8, and regulates the actin cytoskeleton and cell spreading [53-55]. The regulation of mTORC1 is dependent on tuberous sclerosis 1 and 2 proteins (TSC1 and TSC2), which form a stable heterodimer (TSC1:TSC2 complex), of which the TSC2 subunit acts as a GTPase-activating protein for the small GTPase Rheb, which negatively controls Rheb and activates mTOR [50, 56]. The activity of TSC2 is regulated by several protein kinases, such as Akt, extracellular signal-regulated kinase, ribosomal S6 kinase, and AMP-activated protein kinase (AMPK) [57, 58].

AMPK is an important nutrient-sensing protein kinase that plays a critical role in maintaining metabolic homeostasis [59, 60]. Nutrient starvation, caloric restriction, and exercise can lead to ATP depletion and an increase in the AMP to ATP ratio, which triggers the phosphorylation of TSC2 [61,62]. Other research suggests that AMPK can directly inhibit mTORC1 activity through the phosphorylation of its regulatory subunit Raptor [63]. The mechanisms underlying AMPK activation include phosphorylation by its upstream kinases such as liver kinase B1 (LKB1), Ca^2+^/calmodulin-dependent kinase, and transforming growth factor-β-activated kinase 1, and through interaction with regulatory proteins such as kinase suppressor of Ras 2 [64, 65].

When induced by certain stresses, Sesn2 inhibits mTORC1 through the activation of AMPK [10]. Studies have shown that Sesn-deficient cells and tissues exhibit lower AMPK and higher mTORC1 activities under both normal and stress conditions [8, 10, 66]. Although it remains unclear how Sesn2 activates AMPK, Sanli et al. suggested that Sesn2 facilitates the LKB1-dependent phosphorylation of AMPK and functions as a guanine nucleotide dissociation inhibitor for Rag GTPases to control mTORC1 signaling [67, 68]. Sesn2 promotes the metabolic adaptation of cells in response to various insults and mediates genotoxic stress-induced activation of AMPK and the suppression of mTORC1 [10]. Furthermore, as a target gene of p53, Sesn2 acts as a critical link between genotoxic stress, p53, and the mTORC1 signaling pathway. Sesn2 activation inhibits mTORC1 activity, leading to inhibited phosphorylation of S6K and eukaryotic translation initiation factor 4E-binding protein (4E-BP) [10]. By inhibiting the phosphorylation of 4E-BP, Sesn2 enhances its interactions with eIF-4E and inhibits the expression of growth regulatory proteins, such as cyclin D1 and c-Myc [69-71]. More importantly, mTORC1 inhibits autophagy, which plays a critical role in the regulation of cell viability by providing necessary nutrients during starvation and monitoring the health and integrity of organelles, especially mitochondria. Dysfunctional autophagy leads to the accumulation of damaged mitochondria, which produces ROS and thereby triggers oxidative stress and cellular damage [72]. mTORC1 inhibits autophagy through the phosphorylation of ATG1 (ULK1) protein, and ATG13 protein [73]. As an inhibitor of mTORC1, Sesn2 promotes autophagy. The cessation of protein synthesis and the promotion of autophagy are both significant events for the suppression of cell growth during genotoxic stress, and the saved energy can be used for DNA repair, thereby boosting the survival of cells in response to stress insults. Therefore, AMPK/mTORC1 signaling pathway is critical for the role of Sesn2 in controlling cell metabolism and survival under conditions of stress.

More intriguingly, Lee et al. demonstrated that persistent TORC1 activation in *Drosophila* induces *dSesn* through ROS-dependent activation of JNK and FoxO [8]. Furthermore, TORC1 generates ROS to induce *dSesn*; this is prevented by feeding animals vitamin E, an antioxidant. Here, we can see that Sesn2 induced by ROS can scavenge ROS accumulation, suggesting the important role of the TORC1-Sesn2-AMPK-TORC1 feedback loop in maintaining cell metabolism and homeostasis.

## 5. Sesn2 and aging

The hallmarks of aging include the accumulation of aberrant proteins and oxidative stress, dysfunction of cellular metabolism and organs, and defective homeostasis. Moreover, aging is associated with many diseases such as diabetes, cardiovascular diseases, cancer, and neurodegenerative diseases, such as Alzheimer’s disease and Parkinson’s disease. It has been shown that the activation of AMPK, suppression of mTORC1, and stimulation of autophagic signaling are beneficial for extending both lifespan and healthspan [74-78]. Therefore, since Sesn2 can activate AMPK, suppress mTORC1, and stimulate autophagy, it could also slow the aging process.

Lee et al. demonstrated that a loss of *Sesn* in *Drosophila* leads to age-associated pathologies including triglyceride accumulation, mitochondrial dysfunction, muscle degeneration, and cardiac malfunction, all of which could be reversed by pharmacological activation of AMPK or inhibition of TOR [8]. *dSesn*-null adults have a higher level of triglycerides, which were decreased after ectopic expression of *dSesn*. In addition, *dSesn*-null adults show decreased AMPK and increased TOR activities. After feeding *dSesn*-null adults with AMPK-activators such as AICAR (5-aminoimidazole-4-carboxamide 1-β-D-ribofuranoside) or metformin, or the TOR-inhibitor rapamycin, reduced accumulation of triglycerides was found [79, 80]. In addition, the heart function of *dSesn*-null flies is compromised, as demonstrated by arrhythmia and decreased heart rate prevented by feeding AICAR or rapamycin to the flies, which indicated that they had low AMPK activity or high TORC1 activity [8]. *dSesn*-null flies also undergo age-associated muscle degeneration. Twenty-day-old *dSesn*-null flies show degeneration of thoracic muscles with loss of sarcomeric structure, and diffused sarcomere boundaries, which can only be found in old wild-type flies (~90 days). Apart from these signs, muscle cells from 5-day-old *dSesn*-null flies have rounded, enlarged, or disorganized mitochondria, resulting in ROS accumulation, which can be prevented by feeding the flies vitamin E. Importantly, feeding *dSesn*-null flies with AMPK activators and rapamycin also prevents muscle degeneration, suggesting that AMPK/TOR signaling is critical for the prevention of mitochondrial dysfunction and the maintenance of muscular homeostasis during aging. By activating AMPK and inhibiting TORC1, Sesns can reprogram cells to attenuate stress injury by reducing anabolism and enhancing autophagy. Sesns can act as antioxidants to inhibit oxidative damage, promote autophagy, eliminate mitochondrial dysfunction, or inhibit mitochondrial metabolism, which produces ROS. Therefore, Sesns can be thought of as physiological controllers that can attenuate accelerated stress-dependent aging [81]. More importantly, it is worth noting that Sesns are also expressed under normal unstressed conditions, even in the absence of any environmental stress, the *dSesn* knockout mutants show an accelerated aging phenotype [81]. Therefore, Sesns may provide baseline protection to reduce the damage caused by physiological damage, which is an inevitable consequence of basic life processes, such as oxidative respiration and DNA replication.

## 6. Sesn2 and diabetes

As an evolutionarily conserved and nutrient-sensing protein kinase, mTORC1 plays a pivotal role in the regulation of cell metabolism. Chronic mTORC1 activation is induced by nutritional abundance, resulting in enhanced protein and lipid synthesis and inhibited autophagy [82, 83]. By activating S6K, mTORC1 can contribute to insulin resistance by inhibiting the phosphorylation of insulin receptor substrates, and attenuating the PI3K/Akt signaling pathway induced by insulin [84]. On the other hand, liver-specific S6K depletion protects against hepatic steatosis and systemic insulin resistance [84]. Recent studies have suggested that mTORC1 also phosphorylates and activates growth factor receptor-bound protein 10, which has been identified as an inhibitor of PI3K/Akt signaling pathway [85, 86]. Defective autophagy, which can be caused by mTORC1 activation, promotes ER stress and results in insulin resistance [87]. In addition, ER stress leads to the suppression of insulin receptor signaling through hyperactivation of JNK [88]. Therefore, the prolonged activation of mTORC1 contributes to insulin resistance and type II diabetes.

Sesn2, as the inhibitor of mTORC1, is considered to improve insulin resistance. A recent report suggests that exercise induces AMPK and Sesn2 interaction, and leads to increased insulin-sensitivity through autophagy [89]. Sesn2 deficiency enhances obesity-induced insulin resistance and the progression of diabetes. Additionally, Sesn2^-/-^ mice display defective insulin responsiveness and compromised insulin-stimulated PI3K-AKT signaling in the liver [12]. Notably, Sesn2 maintains insulin sensitivity by promoting AMPK activation in the liver, and loss of Sesn2 aggravates hepatosteatosis caused by obesity [12]. Therefore, the regulation of Sesn2 activity may provide an alternative approach for the prevention of insulin resistance, obesity, and diabetes.

## 7. Sesn2 and cancer

Cancer is strongly associated with oxidative stress, metabolic dysregulation, and gene mutation. Cancer cells need to synthesize new proteins and lipids to produce new cells [90]. It has been reported that TORC1 signaling pathway is often activated in human cancers and rapamycin exhibits anti-tumor activity through the inhibition of TORC1 [52]. A recent study suggests that mTORC1 upregulates Golgi protein 73, which promotes the proliferation and migration of hepatocellular carcinoma cells [91]. The mTORC1 inhibitor everolimus confers antitumor activity in vitro, and produces tumor responses in patients with relapsed T-cell lymphoma [92]. Using rapamycin, translation elongation is inhibited, which limits intestinal tumor initiation and growth [93].

Because Sesn2 can suppress TORC1 activation, Sesn2 may confer tumor suppressor activity. Decreased expression of Sesn2 predicts an unfavorable prognosis in colorectal cancer [94]. Sesn2 is shown to be an important regulator of mTORC1 signaling and can inhibit colon carcinogenesis [22]. Sesn2-deficient mouse embryonic fibroblasts were significantly more susceptible to oncogenic transformation than wild type counterparts, suggesting that Sesn2 may have tumor suppressive activity [10]. Sesn2 inhibition leads to the acceleration of A549 lung carcinoma cell growth [30]. These findings indicate that Sesn2 confers tumor a suppressive function. However, Sesn2 is also important for maintaining the viability of cancers under several conditions. As a ROS scavenger, Sesn2 can be activated in cancer cells to attenuate oxidative stress. Moreover, the activation of Sesn2 in tumor cells can activate autophagy, which facilitates tumor cell growth under conditions of limited nutrients and oxygen [95]. Sesn2 positively regulates Akt signaling and survival in human squamous cell carcinoma (SCC) and melanoma cells in response to UVB stress and chemotherapeutics, suggesting that Sesn2 may promote tumorigenesis and chemoresistance in SCC and melanoma [96]. The advantageous or disadvantageous roles of Sesn2 merit further investigation. The results might lead to a novel approach for radiotherapy or chemotherapy in cancers through the regulation of Sesn2 activity.

## 8. Sesn2 and ischemic heart disease

Myocardial ischemia occurs when the heart is subjected to hypoxia. Myocardial ischemia and reperfusion cause bursts of ROS production, leading to cardiac arrhythmia and heart failure [97]. In addition, autophagy is impaired in cardiac ischemia-reperfusion injury and the restoration of autophagosome clearance attenuates reoxygenation-induced cell death [98]. In the Drosophila heart, loss of dSesn function leads to cardiac arrhythmia [8]. Sesn2 also promotes LKB1-mediated AMPK activation in the ischemic heart [13]. By activating autophagy, Sesn2 may confer cardioprotection against ischemia. Since autophagy has a key role in ischemic preconditioning against ischemia [99-102], Sesn2 might confer cardioprotection via this route. Therefore, Sesn2 is a novel therapeutic target to attenuate ROS accumulation and enhance autophagy in response to heart ischemia.

As is widely accepted, aging is accompanied by intensive susceptibility of the myocardium to ischemia/reperfusion injury [103]. This susceptibility of the aged heart is closely related to impaired autophagy and decreased activity of AMPK [104]. Our recent study found that the expression of Sesn2 decreased with aging, and this led to reduced ischemic AMPK activation, significantly impaired downstream glucose uptake, and the oxidation rate. The binding affinity between Sesn2 and AMPK upstream LKB1 is also impaired in aged hearts during ischemia [105]. We found that Sestrin2 prevents age-related intolerance to ischemia and reperfusion injury by modulating substrate metabolism. These findings further confirm that Sesn2 is a potential target that could prevent age-related susceptibility to ischemic heart disease [105, 106].

**Figure 1.**
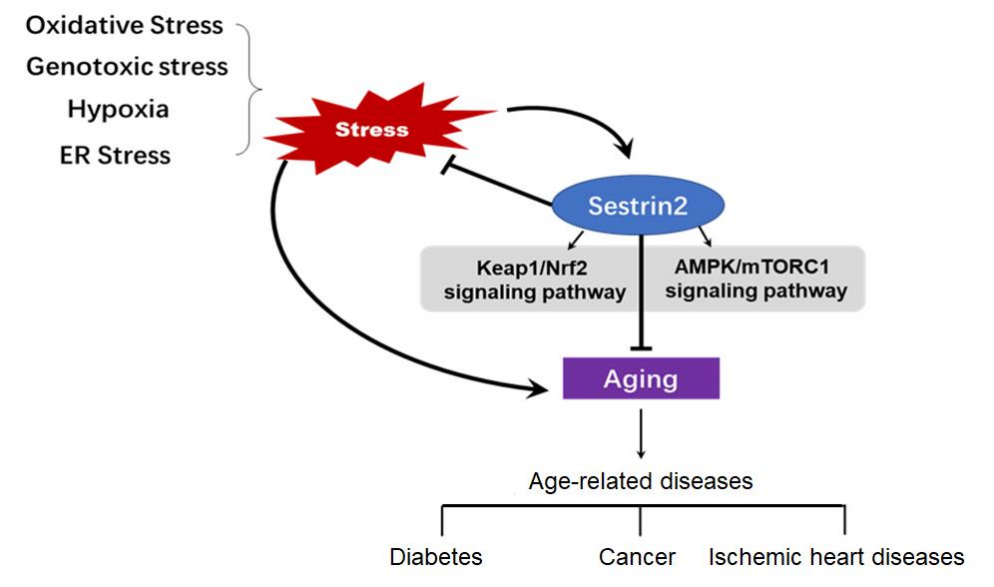
Summary of the merging role of sestrin2 in cell metabolism, cardiovascular and aging-reaged diseases

## 9. Perspectives

Data obtained from animal models indicate that Sesn2 is a promising target for the treatment of cardiovascular diseases in human patients. In this research, we briefly reviewed how Sesn2 may act as a suppressor of aging effects associated with responsive to cell metabolism, stressful stimuli, and age-related cardiovascular diseases (Figure 1). As a target of p53, Sesn2 is considered to be an important regulator of metabolism and aging. Sesn2 can diminish ROS accumulation, and activate autophagy, thus suppressing age-related diseases, obesity, diabetes, neurodegenerative diseases, and cancer. The ability to evaluate whether Sesn2 is activated or inhibited in a given condition and to clarify its functional significance needs to be improved. Sesn2 provides a novel therapeutic target for the prevention of age-related diseases and metabolic disorders. Further research is needed to throw light upon the specific underlying mechanisms by which the functions of Sesn2 are achieved.
